# A rare case of acute angle closure due to spontaneous suprachoroidal haemorrhage secondary to loss of anti-coagulation control: a case report

**DOI:** 10.1186/s12886-018-0857-4

**Published:** 2018-09-14

**Authors:** Ibrahim Masri, Jonathan M. Smith, Nicholas K. Wride, Saurabh Ghosh

**Affiliations:** Sunderland Eye Infirmary, Sunderland, UK

**Keywords:** Suprachoroidal haemorrhage, Acute angle closure, Anticoagulation

## Abstract

**Background:**

Suprachoroidal haemorrhage is a rare complication of either medical anticoagulation treatment or intraocular surgical procedures. Suprachoroidal haemorrhages often have devastating visual outcome despite conservative and/or surgical intervention.

**Case presentation:**

A patient with known Open Angle Glaucoma and Atrial Fibrillation on warfarin presents symptoms and signs suggestive acute angle closure. Examination reveals the underlying cause is a large, macula involving, spontaneous suprachoroidal haemorrhage secondary to loss of anti-coagulation control. Following aggressive medical treatment and surgical intervention, including drainage combined cataract extraction with intraocular lens implant, pars-plana vitrectomy, and external drainage of suprachoroidal haematoma, we managed to preserve the patient’s eye and some of its function.

**Conclusion:**

Spontaneous suprachoroidal haemorrhages are rare complications of loss of anticoagulation control. Our case shows that aggressive treatment in selected cases can offer a relatively good outcome.

**Electronic supplementary material:**

The online version of this article (10.1186/s12886-018-0857-4) contains supplementary material, which is available to authorized users.

## Background

Suprachoroidal haemorrhage is a rare complication of intraocular surgery or trauma. Even more rarely it may be spontaneous. Risk factors include older age, patients on systemic anticoagulation, systemic hypertension, atherosclerosis, age-related macular degeneration and chronic kidney disease. When they occur, Suprachoroidal haemorrhages often have devastating visual outcome despite conservative and/or surgical intervention [1–3].

## Case presentation

We present a case of acute angle closure due to spontaneous suprachoroidal haemorrhage secondary to loss of anti-coagulation control.

A 67-year-old man, who recently returned from a holiday abroad, presented with a one-day history of worsening right visual acuity and 4 day history of increasing right retro-bulbar pain not relieved with simple analgesia.

He had a past medical history of essential tremor managed with Propranolol, Atrial Fibrillation on anticoagulation with Warfarin 4 mg daily – target International Normalised Ratio (INR) 2.5. Possible confusion with his tablets in the week leading up to the start of his symptoms.

Our patient was also known to have normal tension glaucoma (NTG) managed with Latanoprost. He had Selective Laser Trabeculoplasty (SLT) to the right eye 12 months before to improve his intraocular pressure control. His last recorded visual acuity (VA) was 6/6 in both eyes.

**Fig. 1:**
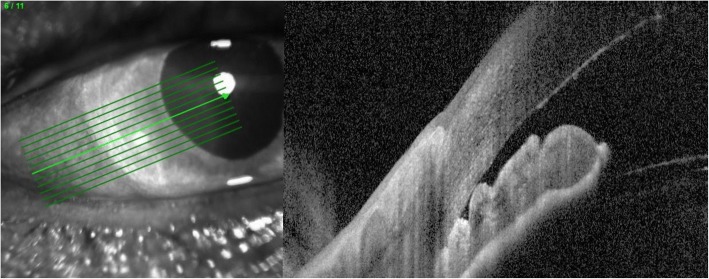
Right eye anterior segment optical coherence tomography (OCT) showing a closed iridocorneal angle

**Fig. 2:**
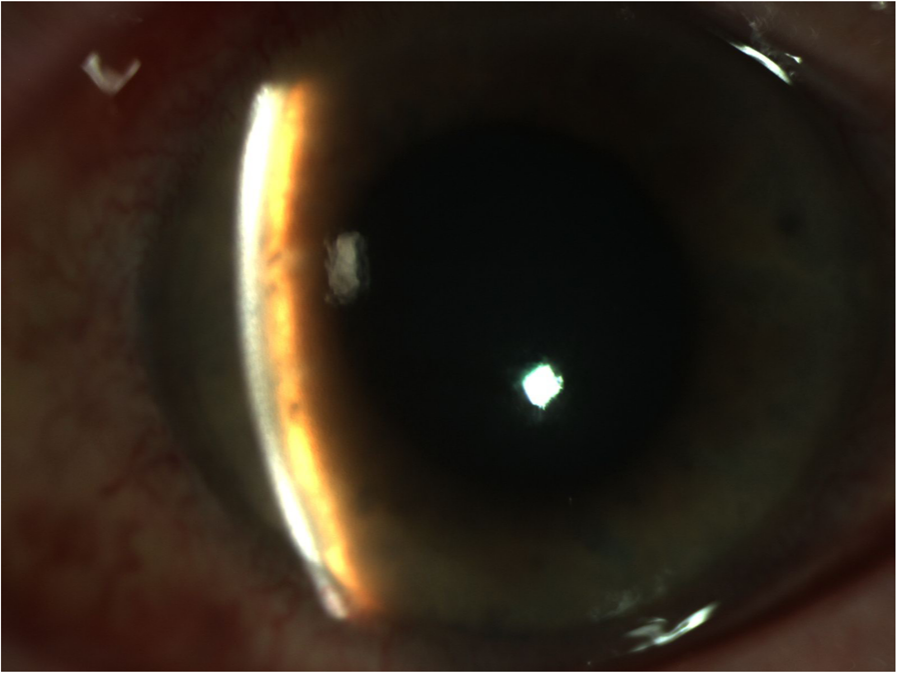
Slit lamp photo of the right eye

**Fig. 3:**
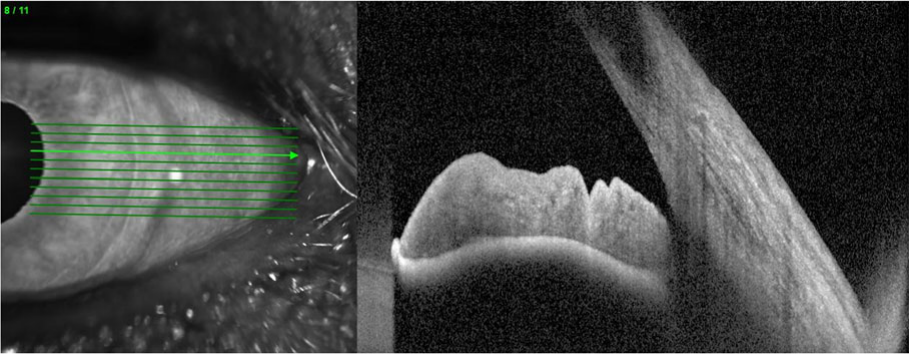
Left eye Anterior Segment OCT showing an open iridocorneal angle

On examination the patient was found to have reduced VA in the right eye 6/12 with an injected conjunctiva, cloudy cornea and a mid-dilated pupil with a very shallow anterior chamber (AC) and closed irido-corneal angle on gonioscopy (Figs. 1 and 2). Fundus exam revealed a large supero-nasal suprachoroidal haemorrhage not involving the macula. His right intra-ocular pressure (IOP) was 42 mmHg. The left eye had a VA of 6/6 with a deep AC and IOP of 12 mmHg (Fig. 3). He was therefore diagnosed with acute angle closure secondary to spontaneous suprachoroidal haemorrhage. His INR measured at > 8. The patient was given 1 mg of Vitamin K to reverse his INR, which quickly came down to 5.1. Advice was taken from the general physicians’ team and no further Vitamin K doses were given.

He was started on maximal topical and systemic IOP lowering treatment including G. Apraclonidine 1% TDS, G. Latanoprost 0.005% ON, G. Brinzolamide/Timolol (Azarga®) and Oral Acetaolamide 250 mg QDS as well as cycloplegia with G. Atropine 1% OD.

**Fig. 4:**
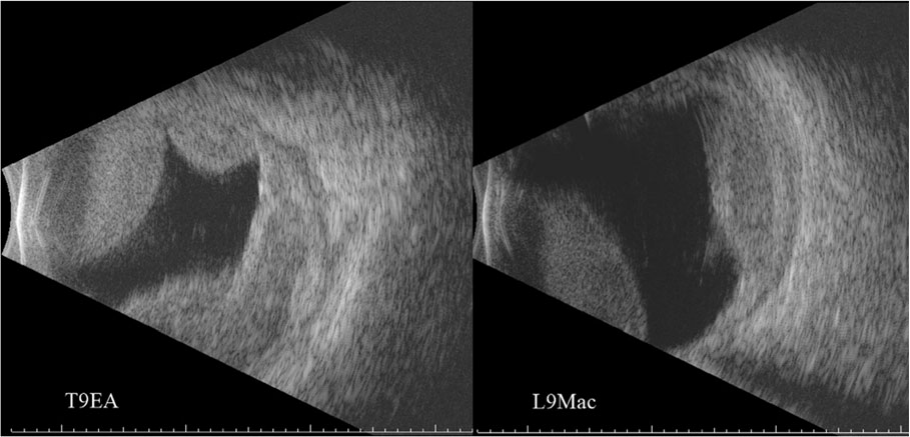
Ultrasound B-Scan of the right eye showing extensive suprachoroidal haemorrhage involving the center of the macula

After 12 h the IOP was 27 mmHg and INR 3.1. But unfortunately, in the following 12 h, the patient had a second bleed, and his IOP went up to 42 mmHg and VA was down to finger counting. There was no view of the fundus due to corneal edema. B-Scan Ultrasound showed an extension of the suprachoroidal haemorrhage, covering 360 degrees and involving the fovea (Fig. 4).

For the next 7 days the patient’s remained on the same medical treatment and his IOP was stable in the high 20s. A decision was taken to perform a combined phacoemulsification and lens implant, pars-plana vitrectomy and suprachoroidal haematoma drainage under general anesthesia. (Additional file 1).


**Additional file 1: Video** of the combined phacoemulsification and lens implant, pars-plana vitrectomy and suprachoroidal haematoma drainage. (AVI 16420 kb)


**Fig. 5:**
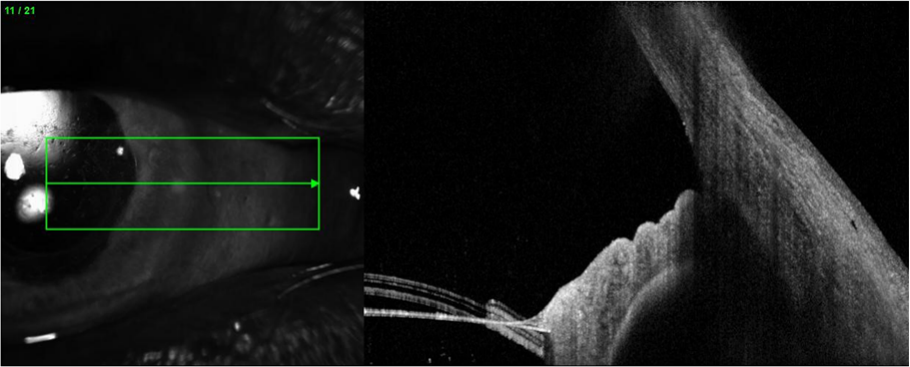
Right eye post operative anterior segment appearance showing a wide open iridocorneal angle

**Fig. 6:**
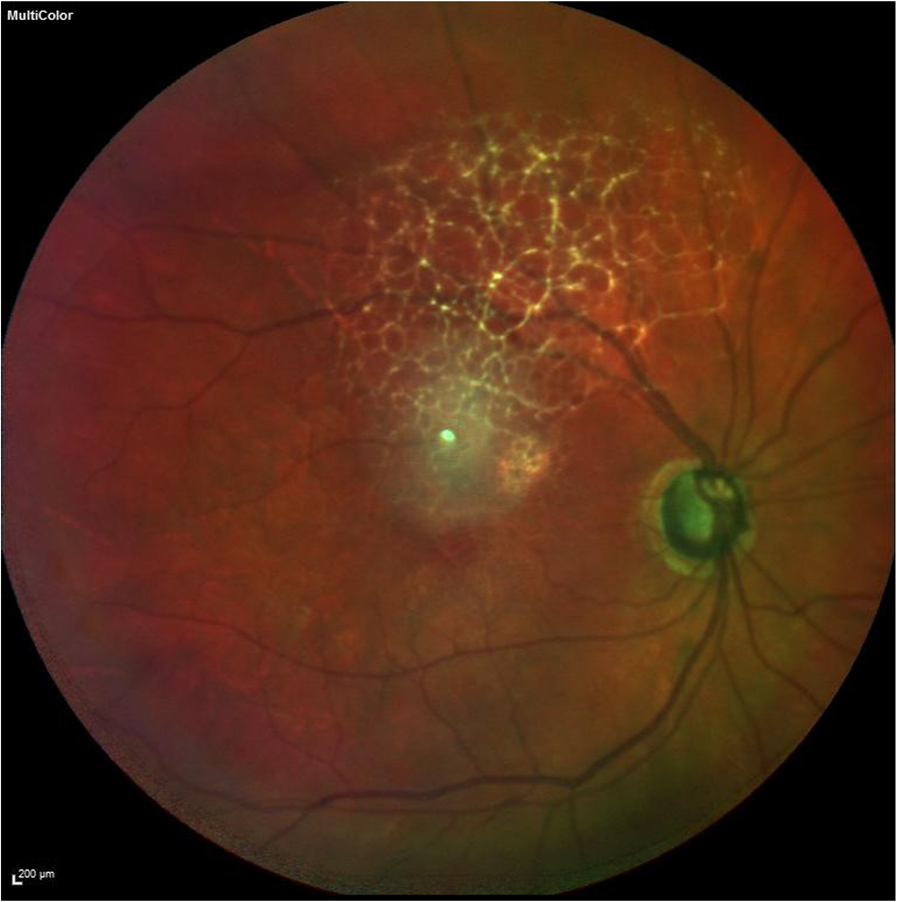
Multicolor photo of the right eye fundus showing a flat retina post operatively

Six weeks post operatively the patient had a wide-open angle with a central IOL and a flat retina (Figs. 5 and 6). Intraocular Pressure without IOP lowering treatment was recorded at 20 mmHg with VA 6/24. He was restarted on IOP lowering topical treatment (G Brinzolamide/Timolol BD).

## Discussion

Subconjunctival haemorrhage, and to a lesser extent spontaneous hyphema are the most common ocular complications of loss of anti-coagulation control [4–6]. Spontaneous suprachoroidal haemorrhage causing acute secondary angle-closure glaucoma is a rare ocular disorder [7]. The proposed mechanism for the angle-closure is the abrupt forward displacement of the lens-iris diaphragm, resulting from a massively detached choroid and retina. A similar mechanism also occurs with serous ciliochoroidal detachments in cases of uveal effusion syndrome, nanophthalmos, scleral buckling procedures, panretinal photocoagulation, central retinal vein obstruction, retinopathy of prematurity, scleritis, pars planitis, Harada’s disease, acquired immunodeficiency syndrome, and arteriovenous fistulas [8]. Posterior uveal melanoma can present with a spontaneous subretinal or intravitreal haemorrhage which can give rise to an acute or chronic angle closure glaucoma [9, 10].

In our patient, loss of coagulation control was due to confusion over two different tablets of the same color. He uses a Monitored Self Dosage (MSD) system, that he organizes himself, when he goes away from home for several days. We postulate that our patient made an error while organizing his MSD system prior to going on holiday. The consequence was inadvertent warfarin overdose, causing loss of anticoagulation control, which led to the spontaneous suprachoroidal haemorrhage in the right eye.

Warfarin belongs to the Coumarin group of Vitamin K antagonists (VKA). It is the most commonly used VKA anticoagulant. Warfarin is usually reversed using systemic administration of vitamin K (commonly a single dose of 0.5 mg – 1 mg). However in cases of life or sight threatening haemorrhages, other agents are available to aided quicker control of coagulation. These are Prothrombin complex concentrate (PCC). Due to the rare nature of such severe bleeds within Ophthalmology practice, the option of PCC use was not considered initially. We can argue that the second haemorrhage could have been avoided if the INR was reversed more aggressively.

## Conclusion

The case describes a rare entity of acute angle closure due to spontaneous suprachoroidal haemorrhage secondary to loss of anti-coagulation control. Most of the previously reported cases of angle-closure glaucoma from massive hemorrhagic retinal or choroidal detachments have failed to respond to medical therapy and needed enucleation or retrobulbar injection treatment for pain [11–13]. Early recognition of this rare entity is vital in preserving the function of the eye. Aggressive medical and surgical treatment with suprachoroidal haematoma drainage offers some chance of preserving the eye and some of its function.

## Abbreviations

AC: Anterior chamber
INR: International Normalised Ratio
IOP: Intra-ocular pressure
MSD: Monitored Self Dosage
NTG: Normal tension glaucoma
OCT: Optical coherence tomography
PCC: Prothrombin complex concentrate
SLT: Selective Laser Trabeculoplasty
VA: Visual acuity
VKA: Vitamin K antagonists
